# Machine learning-based prognostic prediction model of pneumonia-associated acute respiratory distress syndrome

**DOI:** 10.3389/fmed.2025.1582426

**Published:** 2025-07-03

**Authors:** Jing Lv, Juan Chen, Meijun Liu, Xue Dai, Wang Deng

**Affiliations:** Department of Pulmonary and Critical Care Medicine, The First Batch of Key Disciplines on Public Health in Chongqing, Second Affiliated Hospital of Chongqing Medical University, Chongqing, China

**Keywords:** pneumonia, ARDS, machine learning, prediction model, risk factors

## Abstract

**Objective:**

This study aimed to construct a machine learning predictive model for prognostic analysis of patients with p- ARDS.

**Methods:**

In this single-center retrospective study, 230 patients with p- ARDS admitted to the RICU of the second affiliated hospital of Chongqing Medical University from January 2020 to November 2024 were included. Patients were divided into survival group and death group according to the 28-day prognosis results. All patients’ clinical data were first results within 24 h of admission. 20% of the total samples were randomly selected as the test set, and the remaining samples were used as the training set for crossvalidation, and six different models were constructed, including Logistic Regression, Random Forest, NaiveBayes, SVM, XGBoost and Adaboost. The AUC value, AP value, accuracy, sensitivity, specificity, Brier score, and F 1 score were used to evaluate the performance of the models and pick the optimal model. Finally, the SHAP feature importance map was drawn to explain the optimal model.

**Results:**

10 key variables, namely LAR, Lac, pH, age, PO2/FiO2, ALB, BMI, TP, PT, DBIL were screened using the filtration method. The importance ranking of the variables showed that age was the most important variable. Among the six algorithms, the performance of the SVM algorithm is significantly better than that of other algorithms. The AUC, AP, Accuracy, Sensitivity, Specificity, Brier Score, and F1 Scores in the test set were 0.77, 0.67, 0.74, 0.60, 0.81, 0.19, and 0.60, respectively. This indicates the potential value of machine learning models in predicting the prognosis of patients with p- ARDS.

**Conclusion:**

This study developed and visualized a machine learning model constructed based on 10 common clinical features for predicting 28-day mortality in patients with p- ARDS. The model shows good predictive performance and achieves explanatory analysis in combination with SHAP and LIME methods, providing a reliable mortality risk assessment tool for p- ARDS.

## 1 Background

Acute respiratory distress syndrome (ARDS) is a severe lung disease characterized by acute respiratory failure caused by diffuse pulmonary inflammation and edema. It is induced by various pathogenic factors in and out of the lung. The main clinical manifestations included progressive dyspnea, refractory hypoxemia, and diffuse infiltration of the lungs on chest imaging. Studies have shown that 10% of intensive care unit admissions and 23% of mechanically ventilated patients die from ARDS; the mortality rate is 35% and can be as high as 46% in severe ARDS (1). Various predisposing factors, both infectious and noninfectious, can cause direct lung injury through local or systemic inflammation (2). Pneumonia is the leading cause of ARDS (50–80% of all ARDS) (3), poses a major challenge to global health, and ranks among the top 10 causes of death globally (4). Studies have shown significant differences in clinical characteristics and 28-day mortality between patients with direct and indirect ARDS, with a higher mortality rate in patients with direct ARDS (5, 6). Given the high morbidity and mortality of pneumonia-associated ARDS (p- ARDS), it is important to identify in advance which category of p- ARDS patients have a poor prognosis. Early risk stratification provides an important basis for individualized intervention and treatment, helping to reduce mortality in p- ARDS. Widely used scoring systems in the intensive care unit (ICU), such as Acute Physiology and Chronic Health Assessment II (APACHE II) and Sequential Organ Failure Assessment (SOFA) can be used to predict the prognosis of patients with ARDS, but with low specificity (7). Berlin staging had limited predictive power for mortality in ARDS, with an area under the receiver operating characteristic (ROC) curve (AUC) of 0.60 (8). Therefore, the development of novel p- ARDS prediction models is of great clinical value for risk assessment and clinical management optimization. Artificial intelligence machine learning technology has made significant progress in the medical field in recent years. Studies have shown that machine learning has better prediction than traditional statistical analysis (9), and it plays an important role in the diagnosis, risk assessment, mortality prediction, and prognosis analysis of ARDS (10–12). For example, Wu et al. (13), using data from 4,738 patients extracted from the eICU database, constructed a machine model that predicted patients with severe ARDS with an accuracy and AUC of 0.9110, 0.8745, respectively. However, so far there is no machine learning prediction model developed specifically for p- ARDS. Therefore, the clinical risk classification of these patients is not clearly defined, which may make treatment somewhat more challenging. Our study aimed to construct a machine learning prediction model for p- ARDS based on the baseline clinical data of p- ARDS patients and to evaluate the patient characteristics by interpreting the best model to predict the prognosis of p- ARDS patients at an early stage, and to improve the accuracy of the prediction model, to guide clinical decision making.

## 2 Method

### 2.1 Research subjects

In this single-center regression study, 230 patients with p- ARDS admitted to the RICU the second affiliated hospital of Chongqing Medical University from January 2020 to November 2024 were included. The inclusion criteria were as follows: (1) fulfillment of the diagnostic criteria for ARDS in the 2012 Berlin definition (14). (2) Pneumonia is the cause of ARDS. The pneumonia was defined as a new pulmonary infiltrate on chest X-ray or computed tomography and at least one of the following acute lower respiratory infection symptoms: fever, productive cough, purulent expectoration, dyspnea, pleuritic chest pain or focal chest signs on auscultation or abnormal peripheral white cell counts. And it was based on ICD-10 codes J13–J18, which is listed as the primary diagnosis or as comorbidities at admission (15). Exclusion criteria are as follows: (1) Other direct or indirect causes of ARDS, e.g., aspiration of gastric contents, lung contusion, pancreatitis, non-pulmonary sepsis, trauma, burns, and poisoning, etc. (2) under 18 years old, (3) pregnancy, (4) multi-organ failure, (5) failure to obtain informed consent, (6) discharge within 24 h of admission, (7) having incomplete data. Patients were divided into two groups according to their 28-day outcomes, the survival group and the death group.

A total of 230 patients met the inclusion criteria and all the patients were diagnosed with p- ARDS and managed according to international guidelines. They are treated by the same group of doctors, the same group of first-line doctors have roughly the same level, so most patients receive treatment almost the same, which will not have a big difference in the results. All included patients were supported by non-invasive ventilation at the time of RICU admission, the parameters of mechanical ventilation were set to maintain a minimal SPO2 of 93%, a tidal volume around 6 mL/Kg and a respiratory rate lower than 30 per minutes. If these requirements cannot be maintained through non-invasive ventilation, the patient will be intubated and supported with IMV. The IMV was performed with the same target as the non-invasive ventilation. Other adjunctive therapies of ARDS (e.g., Corticosteroid, antibiotic therapy, ECMO, prone positioning, recruitment maneuvers, maintaining of fluid balance, administration of appropriate antimicrobial medications and vasopressors, etc.) were performed at the discretion of the physician in charge (15, 16).

### 2.2 Data

Extract and collect data recorded in the electronic medical record system. Includes patients: (1) demographic characteristics; (2) clinical characteristics (etiology, history of smoking and drinking, past medical history, admission/discharge diagnosis, course of disease, surgery/consultation records, etc.); (3) complications; (4) laboratory indicators (blood gas analysis, procalcitonin (PCT), C-reactive protein (CRP), myocardial injury markers, liver and kidney function, electrolytes, blood routine, coagulation routine) and other variables. The key features were selected from 37 variables by filtering method. All clinical data were first results within 24 h of admission.

### 2.3 Design

In this study, we first use a variety of machine learning algorithms for data classification. These algorithms include: Logistic Regression, Random Forest, NaiveBayes, SVM, XGBoost, and Adaboost. In each training, 80% of the total samples are selected for training, and the remaining samples are verified to ensure that the training samples selected for multiple model algorithms are consistent, thus better comparing multiple models. The optimal hyperparameters of the six ML models were determined by 5-fold cross-validation. When evaluating the performance of the model, receiver operating characteristics (ROC) area under curve (AUC), precision-recall (PR) area under curve (AP), accuracy, sensitivity, specificity, Brier score and F1 score were used. By comparing the AUC, AP, and Brier scores of each model, the model with the highest prediction performance was determined. To facilitate clinical interpretation and application, the SHAP package treats all functions as “contributors” and generates SHAP values, using SHAP values to determine the contribution of each input variable to the model output (17, 18), and SHAP feature importance maps are drawn to interpret the model. In addition, the LIME (Local interpretable model-agnostic explanations) plots were drawn to interpret the prediction results of a single sample and judge the reliability of the model.

### 2.4 Statistical analysis

Patients were divided into two groups based on outcome: those who survived and those who died. Continuous data following a normal distribution are presented as mean ± standard deviations (SD), whereas non-normal data are presented as median and interquartile range. Statistical analyses were performed using SPSS Software (version 27.0, IBM, USA), with chi-square tests and Mann–whitney U tests for categorical and quantitative variables, respectively. Student t test was used for normally distributed continuous variables, and Mann–whitney U test was used for skewed distributed continuous variables. Categorical variables were expressed as percentages or frequencies and compared using the chi-square test. *p* < 0.05 was defined as statistically significant. Variables that were statistically significant (*p* < 0.05) between the survival group and the death group were included in the multivariate logistic regression analysis to identify independent predictors of 28-day mortality. Predictive models were constructed using 6 ML algorithms. All analyses and calculations were performed using Python V3.8.0.

### 2.5 Medical ethics approval

This study was approved by the Ethical Committee of the Second Affiliated Hospital of Chongqing Medical University (No.2022-729). Because of the retrospective nature of this study, the requirement for informed consent was waived by the ethics committee. To ensure confidentiality, all patient information was anonymously recorded.

## 3 Results

### 3.1 Baseline characteristics

A total of 230 patients with p- ARDS were included in the study, with 184 patients in the training set and 46 patients in the validation set during the multi-model comparison. Baseline characteristics of the population are summarized in Table 1.

**Table 1:** Baseline characteristics.

Characteristics	Total (*N* = 230)	Survival group (*N* = 135)	Death group (*N* = 95)	*p*-value
Sex, *n* (%)				0.236
Male	180.00 (78.26)	102.00 (75.55)	78.00 (82.10)	
Female	50.00 (21.74)	33.00 (24.44)	17.00 (17.89)	
Age (years)	68.00 (57.00–76.00)	66.00 (55.00–73.00)	72.00 (61.00–79.00)	<0.001
BMI (Kg/㎡)	23.19 ± 4.04	23.83 ± 3.90	22.28 ± 4.08	0.004
Comorbidities, *n* (%)				
COPD, *n* (%)	9.00 (3.91)	4.00 (2.96)	5.00 (5.26)	0.375
CHD, *n* (%)	30.00 (13.91)	12.00 (8.89)	18.00 (18.95)	0.026
Hypertension, *n* (%)	78.00 (33.91)	42.00 (31.11)	36.00 (37.89)	0.285
Diabetes, *n* (%)	52.00 (22.61)	27.00 (20.00)	25.00 (26.32)	0.260
Laboratory indicators				
WBC (×10^9^/L)	8.89 (6.09–14.13)	9.09 (6.58–14.52)	8.69 (5.09–13.86)	0.077
Lym (×10^9^/L)	0.59 (0.36–0.91)	0.64 (0.43–0.97)	0.47 (0.27–0.83)	0.008
Hb (g/L)	120.00 (101.00–135.00)	123.00 (106.00–138.00)	115.00 (96.00–127.00)	0.012
HCT (%)	35.77 ± 7.02	36.69 ± 90	34.47 ± 7.01	0.018
PLT (×10^9^/L)	176.50 (114.00–257.25)	181.00 (137.00–257.00)	140.00 (82.00–259.00)	0.011
PCT (mg/ml)	0.53 (0.16–2.07)	0.40 (0.14–1.12)	1.06 (0.24–3.85)	<0.001
CTnI (umol/L)	0.02 (0.01–0.05)	0.02 (0.01–0.02)	0.03 (0.02–0.12)	<0.001
Pro-BNP (pg/ml)	767.00 (204.03–2138.01)	442.80 (139.00–1275.80)	1454.40 (628.30–2818.00)	<0.001
D-dimer (ng/ml)	1052.80 (549.30–2865.72)	859.10 (481.70–2490.50)	1553.10 (717.60–4982.70)	0.004
AST (IU/L)	44.50 (26.00–77.50)	43.00 (24.00–67.00)	53.00 (28.00–107.00)	0.049
DBIL (umol/L)	5.80 (3.80–10.90)	5.20 (3.50–8.90)	6.90 (4.30–13.00)	0.009
TP (g/L)	58.32 ± 7.76	59.48 ± 7.70	56.69 ± 7.58	0.007
ALB (g/L)	31.46 ± 4.97	32.28 ± 4.92	30.28 ± 4.84	0.003
BUN (mmol/L)	7.45 (5.23–11.35)	6.67 (4.93–10.49)	8.61 (6.31–12.28)	0.002
Cr (umol/L)	76.25 (57.52–100.32)	72.80 (55.10–87.10)	81.50 (62.60–114.30)	0.007
PT (s)	14.35 (13.30–15.40)	14.00 (13.20–14.90)	14.70 (13.40–16.10)	0.002
pH	7.45 (7.40–7.48)	7.46 (7.42–7.48)	7.42 (7.36–7.47)	<0.001
PO2 /FiO2 (mmHg)	168.50 (122.00–217.00)	180.00 (136.00–224.00)	146.00 (100.00–202.50)	0.002
HCO_3_^−^ (mmol/L)	23.60 (20.90–26.00)	24.10 (21.70–26.10)	22.80 (19.70–25.30)	0.009
Lac (mmol/L)	1.70 (1.10–2.50)	1.50 (1.10–2.10)	2.00 (1.30–3.40)	<0.001
LAR	0.52 (0.36–83)	0.47 (0.34–0.68)	0.67 (0.41–1.22)	<0.001

COPD, Chronic obstructive pulmonary disease; CHD, coronary heart disease; BMI, body mass index; WBC, white blood cell count; Lym, lymphocyte count; Hb, hemoglobin concentration; HCT, hematocrit; PLT, platelet count; PCT, procalcitonin; CTnI, troponin I; Pro-BNP, Pro-Brain Natriuretic Peptide; AST, aspartate aminotransferase; DBIL, direct bilirubin; TP, total protein; ALB, albumin; BUN, blood urea nitrogen; Cr, creatinine; PT, prothrombin time; HCO_3_^−^, bicarbonate; Lac, lactic acid; LAR, Lactate albumin ratio.

The results showed that compared with the survival group, the death group was older (*p* < 0.001) and more likely to have comorbid CHD (*p* = 0.026). Procalcitonin (PCT, *p* < 0.001), troponin I (CTnI, *p* < 0.001), pro-b-type natriuretic peptide (Pro- BNP, *p* < 0.001), D-dimer (D-dimer, *p* = 0.004), aspartate transaminase (AST, *p* = 0.049), direct bilirubin (DBIL, *p* = 0.009), urea nitrogen (BUN, *p* = 0.002), creatinine (CR, *p* = 0.007), prothrombin time (*p* = 0.002), lactate (Lac, *p* < 0.001), and lactatealbumin ratio (LAR, p < 0.001) were higher in the death group; BMI value (*p* = 0.004), lymphocyte number (Lym, *p* = 0.008), hemoglobin content (HB, *p* = 0.012), hematocrit (HCT%, *p* = 0.018), platelet number (PLT, *p* = 0.011), total protein (TP, *p* = 0.007), albumin (ALB, *p* = 0.003), PH (*p* < 0.001), Oxygenation Index (PO2/FiO2, *p* = 0.002), bicarbonate ion (HCO3-, *p* = 0.009) were lower. Variables with statistical significance (*p* < 0.05) between the survival group and the death group were included in the multivariate logistic regression analysis. The results showed that age (OR = 1.041, *p* = 0.002), PH (OR = 0.015, *p* = 0.043), PO2/FiO2 (OR = 0.994, *p* = 0.030), LAR (OR = 2.706, *p* = 0.002), HB (OR = 0.982, *p* = 0.007) were independent predictors of 28-day mortality.

### 3.2 Variable selection

Ten key variables were selected using the filtering method: ‘LAR’, ‘Lac’, ‘pH’, ‘age’, ‘PO2/FiO2’, ‘ALB’, ‘BMI’, ‘TP’, ‘PT’, ‘DBIL’.

### 3.3 Comparison of multi-algorithm models

**Figure 1:**
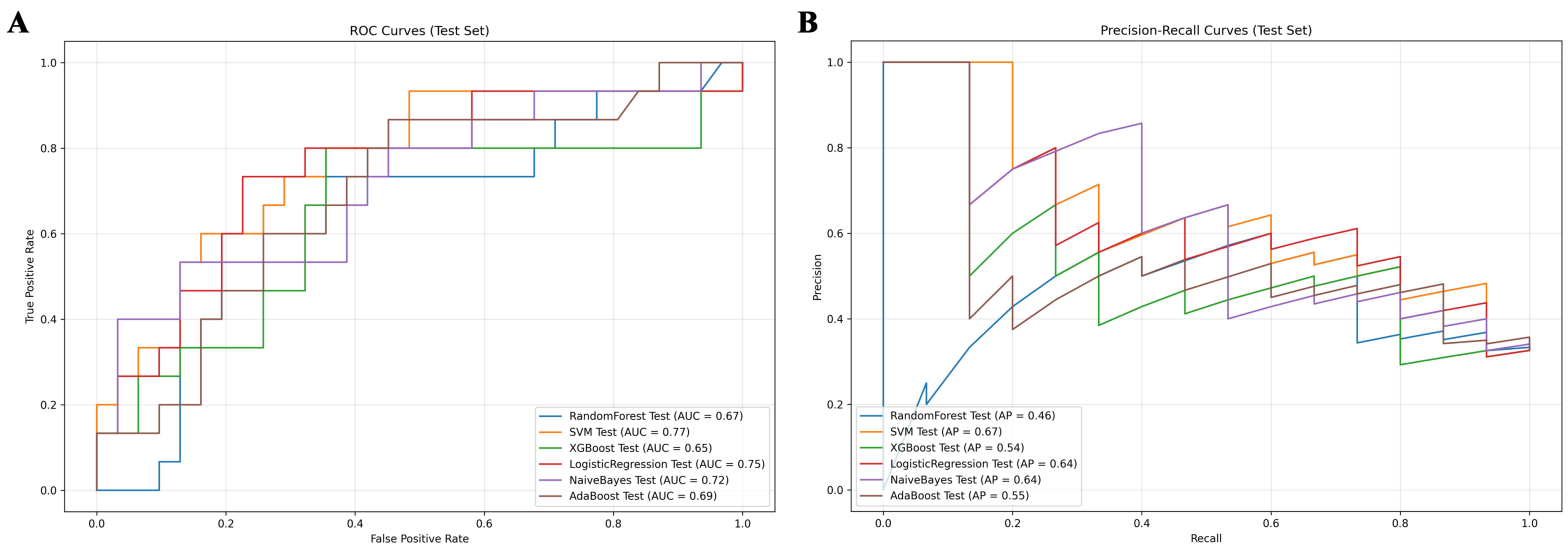
Model comparison chart. **(A)** ROC curves for 6 machine learning models. **(B)** PR curves for 6 machine learning models.

**Figure 2:**
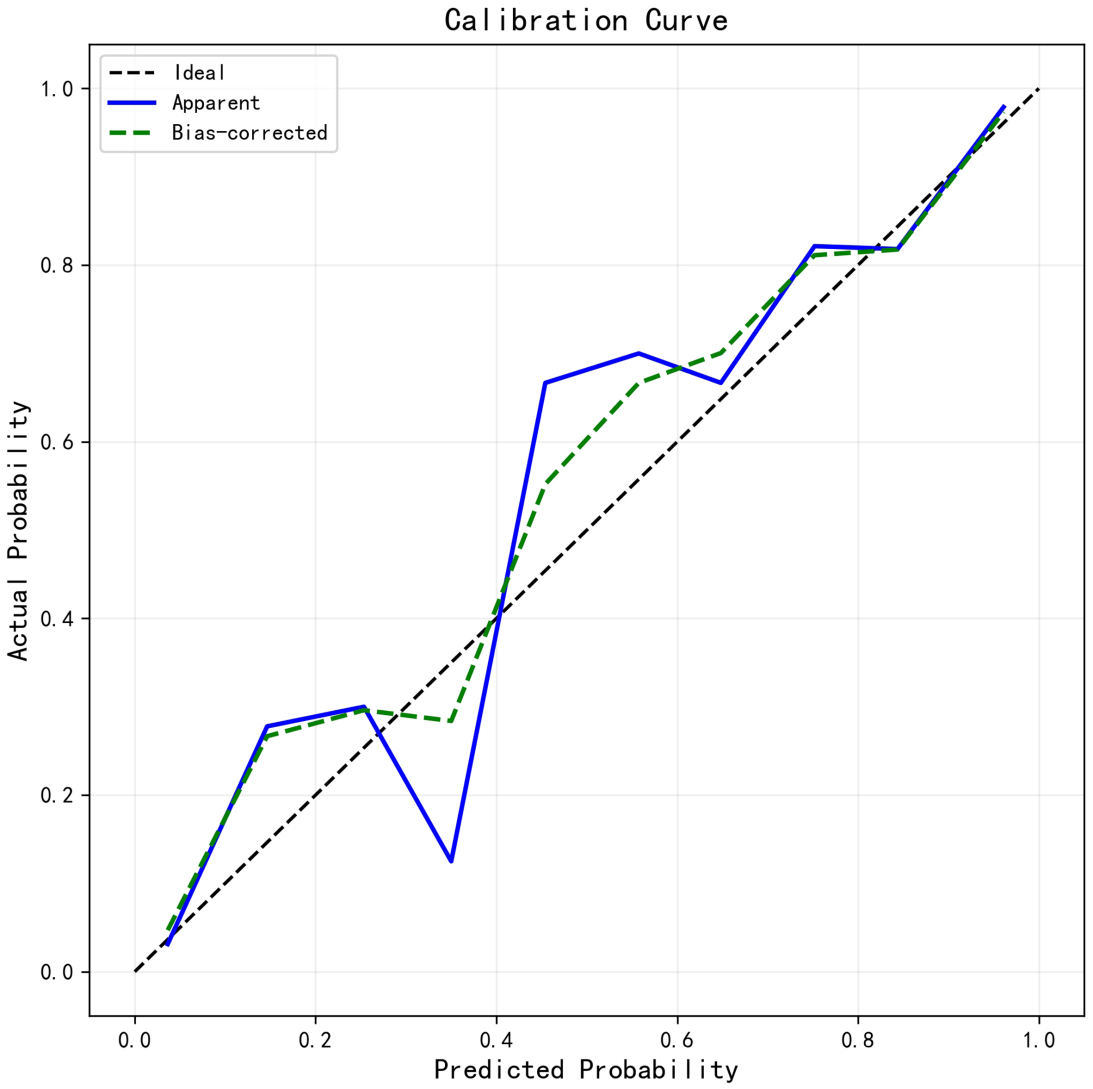
Calibration curve for SVM model.

**Figure 3:**
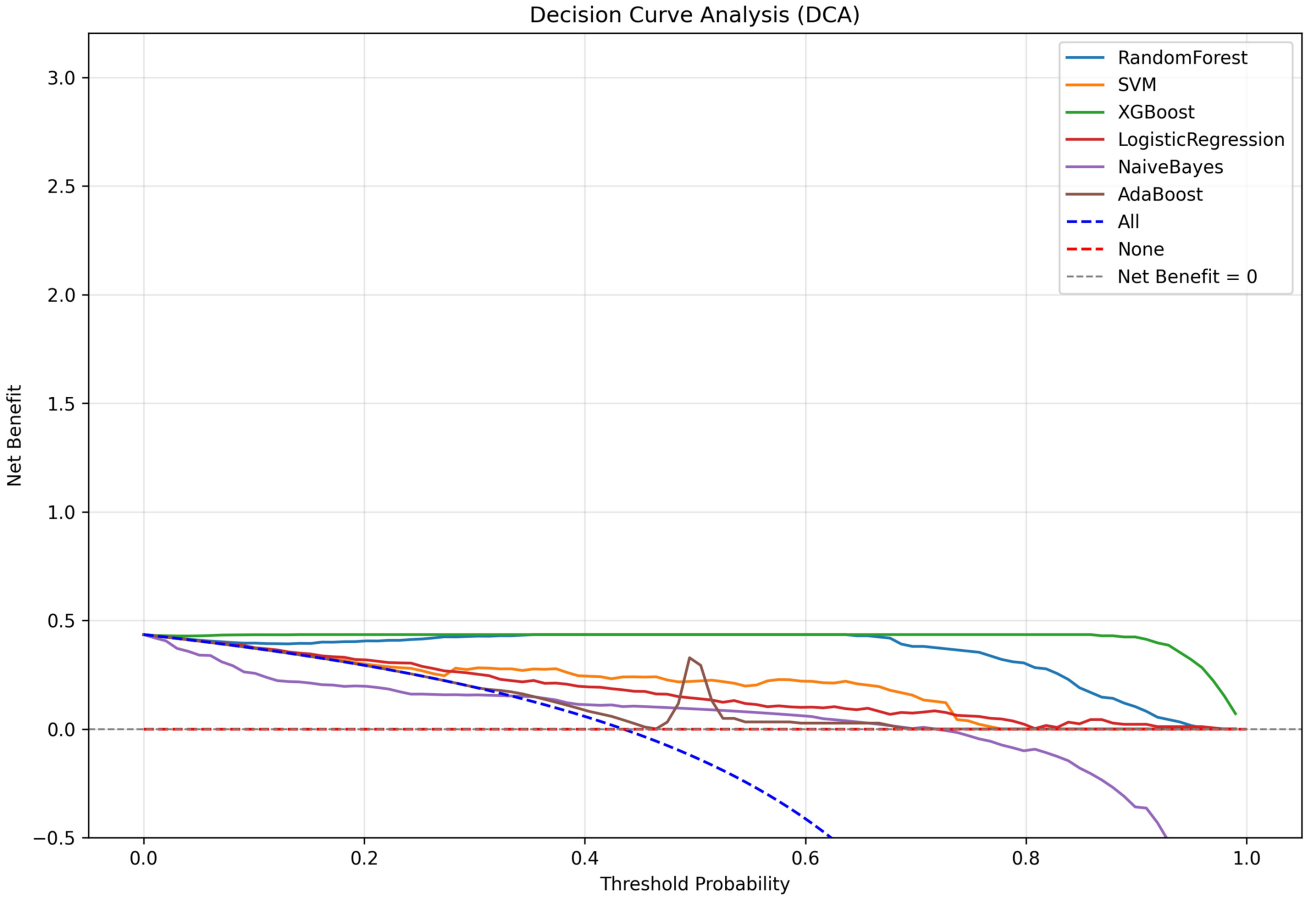
DCA curves of the models.

We developed six ML models: Logistic Regression, Random Forest, NaiveBayes, SVM, XGBoost, and Adaboost, designed to predict 28-day mortality in patients with p- ARDS. After adjusting the hyperparameters, these ML models were trained using the training set, and the performance of these models was evaluated using the test set. The ROC and PR curves for the six models are shown in Figures 1A,B, respectively. In the test set, the AUC values for the Logistic Regression, Random Forest, NaiveBayes, SVM, XGBoost, and Adaboost models were 0.75, 0.67, 0.72, 0.77, 0.65, and 0.69, respectively (Figure 1A), and the AP values were 0.64, 0.46, 0.64, 0.67, 0.54, and 0.69, respectively (Figure 1B). To comprehensively evaluate the performance of the models, the accuracy, sensitivity, specificity, Brier Score, and f1-Score of each model were calculated separately (Table 2). The AUC value and AP value of SVM in the test set are the highest, and the Brier Score is the lowest, which indicates that the model has high discrimination and calibration, so it is the optimal model. The calibration curve for SVM models was illustrated in Figure 2, which demonstrated favorable consistency between predicted probabilities and observed outcomes. The clinical decision curve (DCA) (Figure 3) indicates that the model shows robust net benefits across a wide range of threshold probabilities, suggesting that the model can effectively guide clinical decision-making, help identify the patient groups most in need of intervention, and demonstrate potential clinical benefits.

**Table 2:** Comparison of model metrics.

Model	AUC	AP	Accuracy	Sensitivity	Specificity	Brier Score	F1 Score
Random Forest	0.67	0.46	0.72	0.60	0.77	0.21	0.58
SVM	0.77	0.67	0.74	0.60	0.81	0.19	0.60
XGBoost	0.65	0.54	0.65	0.33	0.81	0.21	0.38
Logistic	0.75	0.64	0.76	0.73	0.77	0.20	0.67
NaiveBayes	0.72	0.64	0.76	0.53	0.87	0.21	0.59
AdaBoost	0.69	0.55	0.70	0.47	0.81	0.23	0.50

### 3.4 Model interpretability

**Figure 4:**
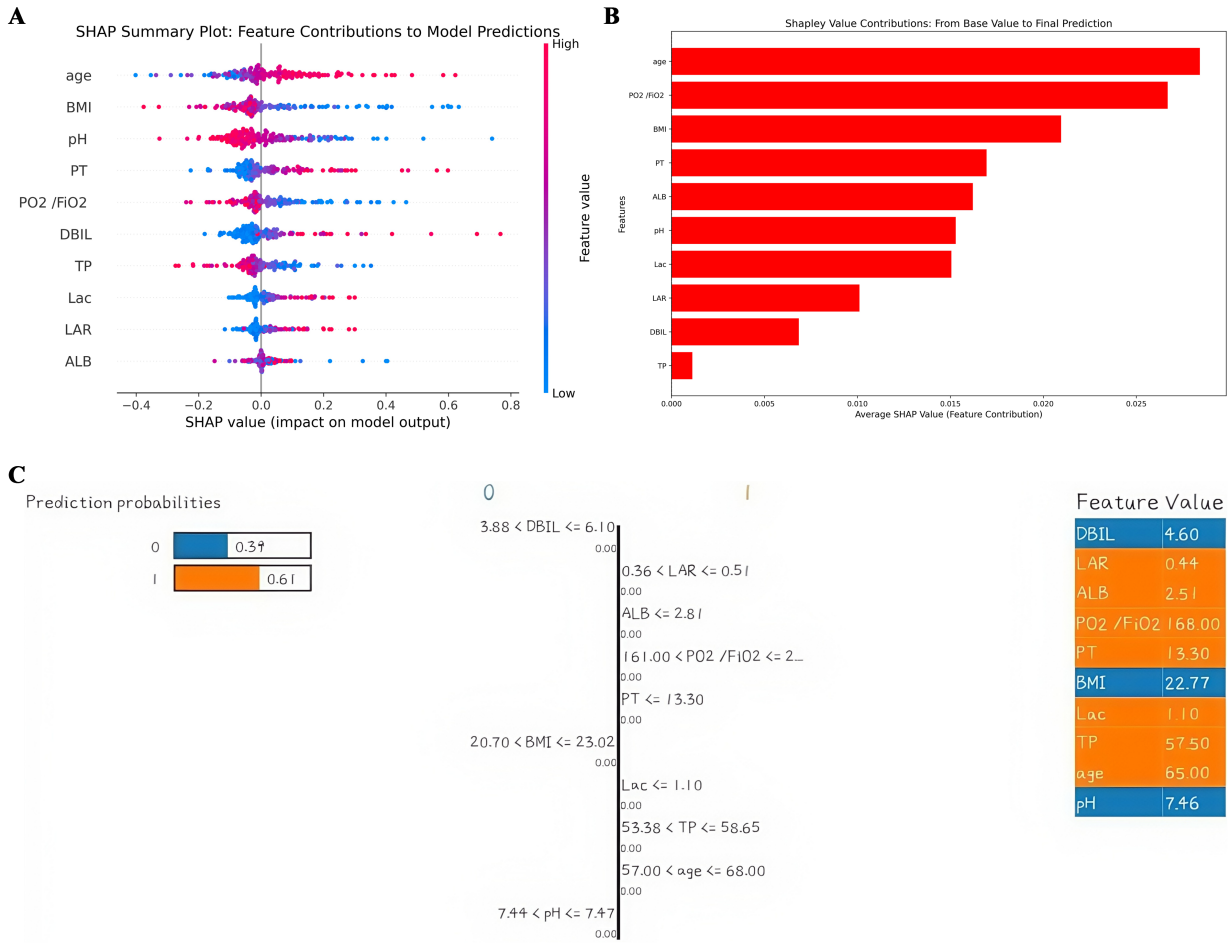
Interpretation of the model. **(A)** SHAP plot of 10 key variables. **(B)** Importance ranking chart of 10 key variables. **(C)** LIME plot of a single sample.

Shapley additive explanations (SHAP) is a method of explaining machine learning predictive models. The SHAP value provides the contribution of each characteristic variable to the results of the prediction model, which helps to understand the decision-making process of the model. To better understand the relationship between the model and the data, we gave a more intuitive interpretation of the best-performing SVM model using SHAP to show how these variables affect 28-day mortality in the model. The bee colony plot in Figure 4A shows the 10 risk factors assessed by SHAP values. Each dot in a row represents a patient, and its color indicates the eigenvalue size-red indicates high values and blue low values. The more right the point is, the greater the positive effect of the feature on the model output is; the more left the point is, the greater the negative effect is. The more scattered the points of the graph, the greater the influence of the variables on the model. Figure 4B shows the important features in this model, where the ranking of the features on the *Y*-axis indicates the importance of the prediction model. Studies have shown a high correlation between age, Oxygenation Index, body mass index and 28-day mortality in patients with p- ARDS. Among them, age is the most important characteristic variable. Furthermore, we provided typical examples of predicted survival and predicted death in Supplementary Figures S1, S2. Interpretation of single-sample predicted outcomes and model reliability judgments were performed using LIME plots (Figure 4C).

## 4 Discussion

Pneumonia is the main cause of ARDS, and p- ARDS has the characteristics of high prevalence and high mortality. It is still challenging to predict its prognosis quickly and accurately in clinical practice. There is an urgent need for an evaluation method with high clinical applicability and universality. Early screening of high-risk patients facilitates the decision-making process of patient management and may improve prognosis. This study is the first attempt to use machine learning methods to construct a clinical predictive model of 28-day mortality in patients with p- ARDS by collecting clinical data. In this retrospective cohort study, we compared the baseline characteristics of the survival group and the death group, and analyzed the differences between the survival group and the death group, multivariate logistic regression analysis showed that age, PH, PO2/FIO2, LAR, and HB were independent predictors of 28-day mortality. And 10 key clinical variables were identified by the filtering method to establish 6 prediction models for mortality in p- ARDS, including Logistic Regression, Random Forest, NaiveBayes, SVM, XGBoost, and AdaBoost. Among them, the SVM model performs the best, with AUC value of 0.77 and the lowest Brier Score, indicating that the model has high discrimination and calibration. The DCA curve shows that the SVM prediction model also has good clinical practicability.

The superior performance of the SVM model in this study may be attributed to the following factors: (1) Its ability to handle high-dimensional spaces: This study involved multiple clinical variables, resulting in a high-dimensional data space. SVM excels at finding the optimal separating hyperplane in high-dimensional spaces, enabling effective data classification. Unlike Logistic Regression, which may be limited when dealing with complex feature correlations, SVM is better suited to handling these high-dimensional clinical data, identifying the boundary that distinguishes related patterns. (2) Adaptability to small sample sizes: This study had a relatively small sample size of only 230 cases. SVM typically performs well in scenarios with small sample sizes. Unlike algorithms such as Random Forest, which may face higher risks of overfitting when sample sizes are small, SVM can identify optimal decision boundaries based on limited samples, thereby performing well in predicting the prognosis of p- ARDS in this study. (3) Advantages in handling nonlinear data: SVM can map low-dimensional nonlinear data to high-dimensional linearly separable spaces using kernel functions (such as radial basis functions), thereby effectively handling such nonlinear relationships. In contrast, linear algorithms like Logistic Regression struggle to handle complex nonlinear relationships (19, 20).

Some important features have been identified in previous studies on the prognosis of p- ARDS. In a risk prediction model that included 75 patients with p- ARDS, the researchers constructed a risk prediction model based on age, Apache II score on Days 3 and 7, CD8 + T cell count, and length of ICU stay with an AUC value of 0.928 (21). Another study included 632 p-ARDS patients admitted to ICU and developed a nomogram containing age, chronic cardiovascular disease, chronic respiratory disease, lymphocyte, ALB, creatinine, D-dimer, and PCT to predict mortality (AUC = 0.808) (22). Consistent with previous findings, the mean age of patients in the p- ARDS death group in this study was 72 years, which was significantly older than that in the survival group. This prognostic difference may be explained by the presence of multiple comorbidities and poor functional status in older patients (23). In the SVM model, age is also the most important feature variable. Notably, several variables in this study were not noticed in previous models, namely BMI, Lar, PH. BMI is a measure of body fatness and nutritional status indicators. Previous studies have suggested a U-shaped or j-shaped association between BMI and mortality in the general population, whereby overweight and obesity are associated with increased risk of all-cause mortality and cardiovascular mortality (24). However, in recent years, researchers have found the ‘obesity paradox’ in patients with heart failure, myocardial infarction, acute coronary syndrome, and chronic obstructive pulmonary disease. In a study of ultra-advanced age (≥80 years) populations, there was an inverse association between BMI and mortality, presenting an inverse j-shaped curve (25). Similarly, the ‘obesity paradox’ phenomenon has been observed in studies of 1-year survival in adult patients with sepsis (26). A similar conclusion was reached in this study, that the BMI value of the survival group was greater than that of the death group (*p* = 0.004), and BMI was also included as an important characteristic variable in the model construction. This may be related to the higher energy reserves, higher tolerance to treatment and better nutritional and immune status of obese patients. This suggests that an active nutrition and conditioning program may be beneficial for patients who anticipate major surgery and may be admitted to the ICU, and that this preparation may enhance their adaptive capacity, as well as their ability to cope with life-threatening conditions, and improve prognosis in the face of critical illness, including ARDS and sepsis. Lactate-to-albumin ratio (LAR) is a new indicator that comprehensively considers individual tissue perfusion metabolism and nutrition, which is mainly suitable for the study of prognosis of critically ill patients. Studies have shown that LAR was an independent predictor of 28-day mortality in patients with ARDS (HR 1.11, 95% CI: 1.06–1.16, *p* < 0.001). The area under the curve (AUC) of LAR in ROC was 70.34% (95% CI: 66.53–74.15%), which provided higher discrimination when compared to lactic acid (AUC = 68.00%, *p* = 0.0007) or albumin (AUC = 63.17%, *p* = 0.002). Kaplan - meier survival analysis showed that 28-day overall mortality (*p* < 0.001) and in-hospital mortality (*p* < 0.001) were significantly higher in patients with ARDS with a high LAR (> cutoff 0.9055) (27). This study also reached similar conclusions that LAR was an independent predictor of 28-day mortality in patients with p- ARDS (OR = 2.706, *p* = 0.002), with higher levels of LAR associated with higher 28-day mortality. Sepsis, kidney failure and impaired respiratory function all disrupt the body’s ability to regulate pH and maintain homeostasis. Studies have shown that increased mortality in intensive care patients is associated with changes in blood pH (28). Meta-analysis of predictors of mortality in severe pneumonia showed that arterial blood PH was associated with severe pneumonia prognosis (29). This study also showed that PH was a risk factor for death in patients with p- ARDS and included PH as a key variable in the prediction model. In addition, another major strength of this study is that fewer clinical indicators are required in the model construction process and are easily available, which means that the medical costs of patients can be saved to a large extent.

This study also enhanced model transparency by combining SHAP and LIME methods to explain the model. SHAP quantifies feature contributions, while LIME provides local explanations of predictive logic. Clinicians can use SHAP values and local explanations of LIME to understand which clinical variables have a greater impact on p- ARDS predictions. By comparing SHAP values across different patients, key factors influencing individual disease severity can also be analyzed. By analyzing patient risk from a pathophysiological perspective using this information, clinicians can assist in assessing disease severity and developing personalized treatment plans. In summary, this model may play an important role in clinical practice. First, in terms of risk stratification, the model can assess patient risk, distinguishing between high-, medium-, and low-risk groups, potentially identifying patients with high mortality risk. This enables healthcare providers to conduct risk communication and provide closer monitoring and early intervention. Second, the model can predict the efficacy of different treatment modalities based on patient characteristics, assisting clinicians in developing personalized treatment plans. Finally, the model can be used to assess patient risk, allocate medical resources reasonably, and improve resource utilization efficiency. However, in clinical practice, model predictions should be combined with clinical judgment. Model predictions provide data support, while clinicians make comprehensive judgments based on their own experience, patient preferences, and other factors to make the best clinical decisions.

Although this study developed and validated an early dynamic prediction model for 28-day mortality in p- ARDS, providing some support for early clinical intervention in high-risk patients, there are still some limitations and more work needs to be done. First, this study is a single-center retrospective analysis with a limited number of patients. Although we grouped the study subjects to assess the stability of the predictive model, the model has not been externally validated. Future studies should validate the model on larger external datasets and evaluate its performance in multicenter prospective studies to demonstrate its generalizability. Second, this study focused on predicting p- ARDS mortality based on initial conditions at admission, when treatment had not yet been fully initiated. Initial clinical variables better reflect the natural course of the disease. To avoid confounding bias introduced by treatment interventions, treatment variables were not included. However, this may have overlooked the potential impact of treatment on outcomes, leading to some bias in the model. Future studies should consider refining the inclusion of treatment variables to assess their corrective effect on model predictions. Additionally, the type and cause of pneumonia may lead to different outcomes for ARDS patients. However, due to insufficient data, we were unable to conduct further subgroup analyses. Fourth, our model uses easily accessible clinical variables, enhancing its practicality. However, in different healthcare settings, factors such as differences in equipment and resources, staff expertise and operational standards, and patient cooperation may pose challenges to the continuous collection of these variables. Therefore, future studies should investigate the data collection capabilities of healthcare facilities at different levels, propose standardized data collection processes or alternative indicator schemes, and ensure the model’s applicability across different scenarios. Finally, imaging data were not collected. Simple laboratory test results are less detailed than comprehensive imaging studies and laboratory data. However, using only these data can help patients save on prediction costs and medical expenses, and integrate more measures into the diagnostic system to achieve personalized treatment.

## 5 Conclusion

In conclusion, this study developed and visualized a convenient and economical prediction model for predicting 28-day mortality in patients with p- ARDS. The machine learning prediction model consisting of 10 common clinical features had satisfactory prediction performance, which indicates the potential value of machine learning models in predicting the prognosis of patients with p- ARDS, may enable clinicians to better predict mortality risk. This study combines machine learning with SHAP and LIME to explain the model in depth while also facilitating the optimization of the model. The model can also be applied to risk prediction of other diseases and provide better interpretation. Future multicenter prospective studies with larger sample sizes are needed to confirm our results and validate or improve our predictive models.

## Data Availability

The raw data supporting the conclusions of this article will be made available by the authors, without undue reservation.
