# Supplementary material for: Machine learning-based prognostic prediction model of pneumonia-associated acute respiratory distress syndrome
Source: Front Med (Lausanne). 2025 Jul 3;12:1582426. doi: 10.3389/fmed.2025.1582426 (PMC12268496; doi:10.3389/fmed.2025.1582426)
Supplement: Supplementary file 1 [file Data_Sheet_1.pdf]

```
import pandas as pd
df=pd.read_excel('分析数据 1.0(新).xlsx')
import pandas as pd
import numpy as np
from sklearn.model_selection import train_test_split
from sklearn.preprocessing import StandardScaler, LabelEncoder
from sklearn.ensemble import RandomForestClassifier
from sklearn.svm import SVC
from xgboost import XGBClassifier
from sklearn.metrics import classification_report, accuracy_score, roc_auc_score, confusion_matrix, f1_score
import matplotlib.pyplot as plt
import seaborn as sns

# 数据预处理
# 编码 sex（如果是非数字类型的话）
le = LabelEncoder()
df['sex'] = le.fit_transform(df['sex'])

# 特征和目标分离
X = df.drop(columns=['outcome'])  # 假设 '身高' 和 'number' 不参与训练
y = df['outcome']

# 特征与目标变量相关性分析
# 将数据合并以便计算相关性
data = pd.concat([X, y], axis=1)
correlation_matrix = data.corr()

# 目标变量与其他特征的相关性排序
target_corr = correlation_matrix['outcome'].drop('outcome').abs().sort_values(ascending=False)

# 显示相关性前 10 的特征
top_features = target_corr.head(10).index.tolist()
print("相关性较强的特征：", top_features)

# 可视化相关性热力图
plt.figure(figsize=(12, 8))
sns.heatmap(data[top_features + ['outcome']].corr(), annot=True, fmt='.2f', cmap='coolwarm')
plt.title("Top 10 Features Correlation with Outcome")
plt.show()

# 选择相关性较强的特征作为输入
X_selected = X[top_features]
```

```
# 数据标准化
scaler = StandardScaler()
X_scaled = scaler.fit_transform(X_selected)

# 数据集划分（80%训练集，20%测试集）
X_train, X_test, y_train, y_test = train_test_split(X_scaled, y, test_size=0.2, random_state=42)
```

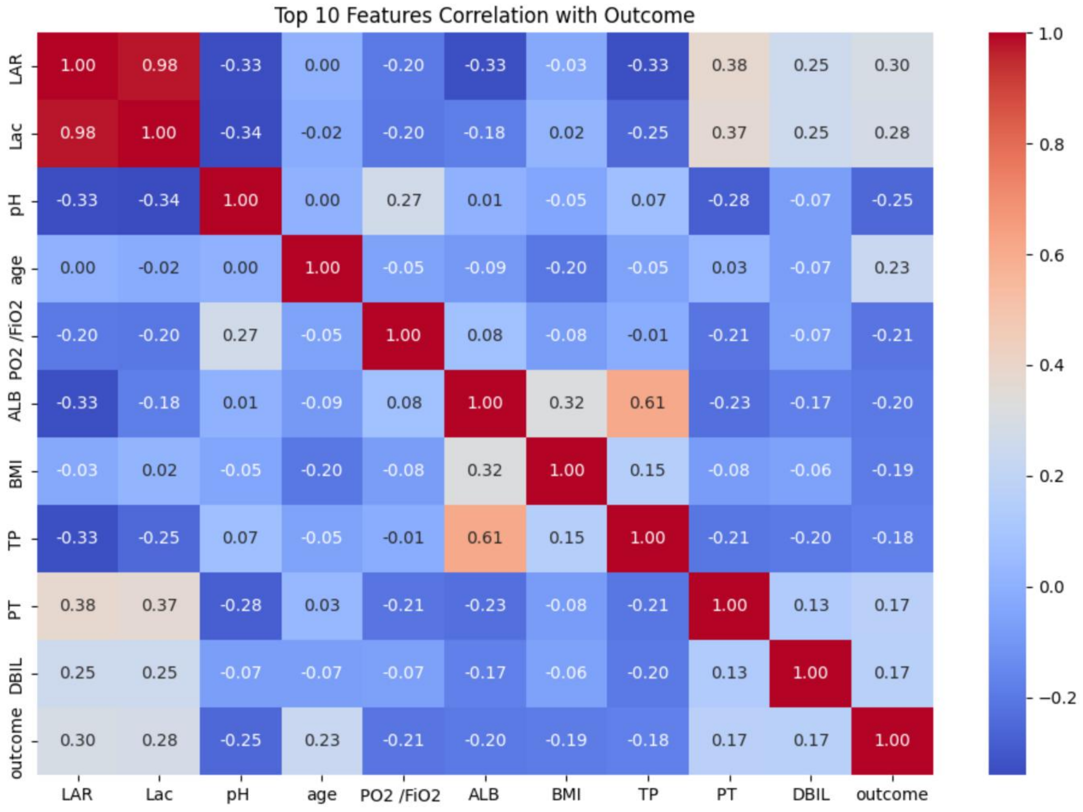


```
from sklearn.model_selection import GridSearchCV
from sklearn.metrics import roc_curve, auc, accuracy_score, f1_score, roc_auc_score
import matplotlib.pyplot as plt
import pandas as pd

# 定义模型和超参数网格
from sklearn.ensemble import RandomForestClassifier
from sklearn.svm import SVC
from xgboost import XGBClassifier
from sklearn.linear_model import LogisticRegression
from sklearn.neighbors import KNeighborsClassifier
from sklearn.naive_bayes import GaussianNB
from sklearn.ensemble import AdaBoostClassifier
from sklearn.tree import DecisionTreeClassifier

models = {
    'RandomForest': RandomForestClassifier(),
    'SVM': SVC(probability=True),
```

```
        'XGBoost': XGBClassifier(),
        'LogisticRegression': LogisticRegression(),
        'NaiveBayes': GaussianNB(),
        'AdaBoost': AdaBoostClassifier(),
}

param_grids = {
        'RandomForest': {
                'n_estimators': [100, 200],
                'max_depth': [10, 20],
                'min_samples_split': [2, 5],
                'min_samples_leaf': [1, 2],
        },
        'SVM': {
                'C': [0.1, 1, 10],
                'kernel': ['linear', 'rbf'],
                'gamma': ['scale', 'auto']
        },
        'XGBoost': {
                'n_estimators': [100, 200],
                'learning_rate': [0.01, 0.1],
                'max_depth': [3, 6]
        },
        'LogisticRegression': {
                'C': [0.1, 1, 10],
                'solver': ['liblinear', 'saga']
        },
        'AdaBoost': {
                'n_estimators': [50, 100],
                'learning_rate': [0.01, 0.1, 1]
        },

}import matplotlib.pyplot as plt
from sklearn.metrics import roc_curve, auc, precision_recall_curve, average_precision_score
import numpy as np

import matplotlib.pyplot as plt
from sklearn.metrics import roc_curve, auc, precision_recall_curve, average_precision_score
import numpy as np

#  训练和评估模型
train_roc_data = {}
test_roc_data = {}
train_pr_data = {}
```

```python
test_pr_data = {}

# 定义绘制函数
def plot_roc_curves(train_roc_data, test_roc_data):
    plt.figure(figsize=(12, 8))
    # 绘制随机猜测线
    plt.plot([0, 1], [0, 1], color='gray', linestyle='--', label='Random Guess')

    # 训练集 ROC 曲线
    for model_name, (train_fpr, train_tpr, train_auc) in train_roc_data.items():
        plt.plot(train_fpr, train_tpr, label=f'{model_name} Train (AUC = {train_auc:.2f})',
linestyle='--')
    plt.title('ROC Curves (Train Set)')
    plt.xlabel('False Positive Rate')
    plt.ylabel('True Positive Rate')
    plt.legend(loc='lower right')
    plt.grid(alpha=0.3)
    # 保存高分辨率图像
    plt.savefig('roc_train_set.png', dpi=300)
    plt.show()

    plt.figure(figsize=(12, 8))
    # 测试集 ROC 曲线
    for model_name, (test_fpr, test_tpr, test_auc) in test_roc_data.items():
        plt.plot(test_fpr, test_tpr, label=f'{model_name} Test (AUC = {test_auc:.2f})',
linestyle='-')
    plt.title('ROC Curves (Test Set)')
    plt.xlabel('False Positive Rate')
    plt.ylabel('True Positive Rate')
    plt.legend(loc='lower right')
    plt.grid(alpha=0.3)
    # 保存高分辨率图像
    plt.savefig('roc_test_set.png', dpi=300)
    plt.show()

def plot_pr_curves(train_pr_data, test_pr_data):
    plt.figure(figsize=(12, 8))
    # 训练集 PR 曲线
    for model_name, (train_precision, train_recall, train_ap) in train_pr_data.items():
        plt.plot(train_recall, train_precision, label=f'{model_name} Train (AP = {train_ap:.2f})',
linestyle='--')
    plt.title('Precision-Recall Curves (Train Set)')
    plt.xlabel('Recall')
    plt.ylabel('Precision')
```

```
    plt.legend(loc='lower left')
    plt.grid(alpha=0.3)
    # 保存高分辨率图像
    plt.savefig('pr_train_set.png', dpi=300)
    plt.show()

    plt.figure(figsize=(12, 8))
    # 测试集 PR 曲线
    for model_name, (test_precision, test_recall, test_ap) in test_pr_data.items():
        plt.plot(test_recall, test_precision, label=f'{model_name} Test (AP = {test_ap:.2f})',
linestyle='-')
    plt.title('Precision-Recall Curves (Test Set)')
    plt.xlabel('Recall')
  plt.ylabel('Precision')
    plt.legend(loc='lower left')
    plt.grid(alpha=0.3)
    # 保存高分辨率图像
    plt.savefig('pr_test_set.png', dpi=300)
    plt.show()

def plot_dca_curves(models, X_train, y_train, thresholds=np.linspace(0, 1, 100)):
    import numpy as np
    import matplotlib.pyplot as plt

    plt.figure(figsize=(12, 8))

    for model_name, model in models.items():
        model.fit(X_train, y_train)
        # 获取预测概率
        prob = model.predict_proba(X_train)[:, 1]

        net_benefit = []
        for threshold in thresholds:
     # 根据阈值计算净收益
            predictions = (prob >= threshold).astype(int)
            tp = np.sum((predictions == 1) & (y_train == 1))
            tn = np.sum((predictions == 0) & (y_train == 0))
            fp = np.sum((predictions == 1) & (y_train == 0))
            fn = np.sum((predictions == 0) & (y_train == 1))

            # 避免除零错误
            if tp + fn > 0 and fp + tn > 0:
                net_benefit.append((tp / len(y_train)) - ((fp / len(y_train)) * (threshold / (1 -
threshold))))
```

```
            else:
                net_benefit.append(0)

        plt.plot(thresholds, net_benefit, label=f'{model_name}')

    # 添加"all"（假设所有人都接受干预）
    all_net_benefit = [np.mean(y_train) - (threshold / (1 - threshold)) * (1 - np.mean(y_train))
for threshold in thresholds]
    plt.plot(thresholds, all_net_benefit, label='All', linestyle='--', color='blue')

    # 添加"none"（假设无人接受干预）
    none_net_benefit = [0 for _ in thresholds]
    plt.plot(thresholds, none_net_benefit, label='None', linestyle='--', color='red')

    # 图像设置
    plt.axhline(0, color='gray', linestyle='--', linewidth=1, label='Net Benefit = 0')
    plt.xlabel('Threshold Probability')
    plt.ylabel('Net Benefit')
    plt.title('Decision Curve Analysis (DCA)')
    plt.ylim(-0.5, None)  # 设置 y 轴下限为 -0.5，上限自动调整
    plt.legend()
    plt.grid(alpha=0.3)

    # 保存高分辨率图像
    plt.savefig('dca_curve.png', dpi=300)
    plt.show()


metrics_results=[]
# 训练模型并评估
for model_name, model in models.items():
    print(f"Optimizing {model_name}...")

    # 获取参数网格并训练模型
    param_grid = param_grids.get(model_name, None)
    if param_grid:
        grid_search = GridSearchCV(model, param_grid, cv=5, scoring='accuracy', n_jobs=-1,
verbose=1)
        grid_search.fit(X_train, y_train)
        best_model = grid_search.best_estimator_
        best_params = grid_search.best_params_
        print(f"Best Params for {model_name}: {best_params}")
    else:
        best_model = model
```

```
        best_model.fit(X_train, y_train)

    # 预测结果（测试集和训练集）
    y_test_pred = best_model.predict(X_test)
    y_train_pred = best_model.predict(X_train)

    # 计算概率（测试集和训练集）
    if hasattr(best_model, "predict_proba"):
        y_test_prob = best_model.predict_proba(X_test)[:, 1]
        y_train_prob = best_model.predict_proba(X_train)[:, 1]
    else:
   y_test_prob = best_model.decision_function(X_test)
        y_train_prob = best_model.decision_function(X_train)
        y_test_prob = (y_test_prob - y_test_prob.min()) / (y_test_prob.max() - y_test_prob.min())
        y_train_prob = (y_train_prob - y_train_prob.min()) / (y_train_prob.max() - y_train_prob.min())
    tn, fp, fn, tp = confusion_matrix(y_test, y_test_pred).ravel()
    sensitivity = tp / (tp + fn)  # 灵敏度（召回率）
    specificity = tn / (tn + fp)  # 特异度
    brier_score = brier_score_loss(y_test, y_test_prob)  # Brier 分数
    # Metrics
    metrics = {
            'Model': model_name,
            'Train Accuracy': accuracy_score(y_train, y_train_pred),
            'Test Accuracy': accuracy_score(y_test, y_test_pred),
            'Train F1 Score': f1_score(y_train, y_train_pred),
            'Test F1 Score': f1_score(y_test, y_test_pred),
            'Train ROC AUC': roc_auc_score(y_train, y_train_prob),
            'Test ROC AUC': roc_auc_score(y_test, y_test_prob),
            'Train PR AUC': average_precision_score(y_train, y_train_prob),
            'Test PR AUC': average_precision_score(y_test, y_test_prob),
            'Sensitivity': sensitivity,
            'Specificity': specificity,
            'Brier Score': brier_score
        }


        # Print metrics to console
    print("\nEvaluation Metrics:")
    for metric, value in metrics.items():
        if metric != 'Model':
            print(f"{metric}: {value:.4f}")
    metrics_results.append(metrics)
```

```
    # 计算并存储 ROC 曲线数据
    test_fpr, test_tpr, _ = roc_curve(y_test, y_test_prob)
    train_fpr, train_tpr, _ = roc_curve(y_train, y_train_prob)
    test_roc_data[model_name] = (test_fpr, test_tpr, auc(test_fpr, test_tpr))
    train_roc_data[model_name] = (train_fpr, train_tpr, auc(train_fpr, train_tpr))

    # 计算并存储 PR 曲线数据
    test_precision, test_recall, _ = precision_recall_curve(y_test, y_test_prob)
    train_precision, train_recall, _ = precision_recall_curve(y_train, y_train_prob)
    test_pr_data[model_name] = (test_precision, test_recall, average_precision_score(y_test,
y_test_prob))
    train_pr_data[model_name] = (train_precision, train_recall,
average_precision_score(y_train, y_train_prob))

# 将结果保存为 CSV 文件
results_df = pd.DataFrame(metrics_results)
results_df.to_csv('model_evaluation_metrics.csv', index=False)
print("Metrics have been saved to 'model_evaluation_metrics.csv'.")

# 绘制训练集和测试集的对比 ROC 曲线
plot_roc_curves(train_roc_data, test_roc_data)

# 绘制训练集和测试集的对比 PR 曲线
plot_pr_curves(train_pr_data, test_pr_data)

# 绘制 DCA 决策曲线
plot_dca_curves(models, X_train, y_train)
import shap
import matplotlib.pyplot as plt
import numpy as np

# 假定 `model` 是经过训练的 XGBoost 模型
model = models['SVM']

# SHAP Explainer
explainer = shap.KernelExplainer(model.predict, shap.kmeans(X_train, 10))  # 使用 k-means
近似减少计算量
shap_values = explainer(X_train)
X_train = scaler.inverse_transform(X_train)
# ---------------------
# 1. Summary Plot with SHAP Values
# ---------------------
plt.figure(figsize=(12, 8))
shap.summary_plot(shap_values, X_train, feature_names=top_features, show=False)
```

```
plt.title("SHAP Summary Plot: Feature Contributions to Model Predictions", fontsize=14)
plt.tight_layout()
plt.savefig("shap_summary_plot.png", dpi=500, bbox_inches="tight")
plt.show()

# ---------------------
# 2. Shapley Value Explanation (Concept and Visualization)
# ---------------------
# Draw a concept visualization of Shapley Values
plt.figure(figsize=(12, 8))
values = shap_values.values.mean(axis=0)    # Compute the mean of SHAP values
sorted_indices = np.argsort(values)    # Sort indices
sorted_values = values[sorted_indices]
sorted_features = [top_features[i] for i in sorted_indices]

# Plot the contribution graph
colors = ['red' if val > 0 else 'blue' for val in sorted_values]
plt.barh(sorted_features, sorted_values, color=colors)
plt.axvline(0, color='black', linestyle='--', linewidth=1)
plt.title("Shapley Value Contributions: From Base Value to Final Prediction", fontsize=14)
plt.xlabel("Average SHAP Value (Feature Contribution)", fontsize=12)
plt.ylabel("Features", fontsize=12)
plt.tight_layout()
plt.savefig("shap_concept_plot.png", dpi=500, bbox_inches="tight")
plt.show()
import shap
import matplotlib.pyplot as plt
import numpy as np



# 使用模型进行预测
y_pred = model.predict(X_test)    # 二分类模型的预测类别结果

# 创建 SHAP explainer 并计算 SHAP 值
explainer = shap.KernelExplainer(model.predict, shap.kmeans(X_test, 10))    # 使用 k-means 近似减少计算量
shap_values = explainer(X_test)
X_test = scaler.inverse_transform(X_test)
# 找到预测为 0 和 1 的样本索引
survived_indices = np.where(y_pred == 0)[0]
death_indices = np.where(y_pred == 1)[0]
# 设置图像的大小
fig_size = (12, 8)    # 你可以根据需要调整宽度和高度
```

```
# 绘制预测为 0 的样本 SHAP force plot
print("预测为 0（存活）的样本:")
for i in survived_indices[:5]:  # 仅绘制前 5 个样本
    shap.force_plot(
    explainer.expected_value,  # Remove [0] here
    shap_values.values[i],
    X_test[i],
    feature_names=top_features,
    matplotlib=True
   )
    plt.show()
    plt.savefig(f"survived_sample_{i}.png")  # 保存图像


# 绘制预测为 1 的样本 SHAP force plot
print("预测为 1（死亡）的样本:")
for i in death_indices[:5]:  # 仅绘制前 5 个样本
    shap.force_plot(
        explainer.expected_value,
        shap_values.values[i],
        X_test[i],
         feature_names=top_features,
        matplotlib=True
    )
    plt.show()
    plt.savefig(f"death_sample_{i}.png")  # 保存图像
    import shap
    import matplotlib.pyplot as plt
    import numpy as np
    from sklearn.inspection import permutation_importance
    from lime.lime_tabular import LimeTabularExplainer
    from sklearn.svm import SVC  # 假设是 SVM 分类模型

    # 假设 'models' 中有一个 SVM 模型
    model = models['SVM']

    # ---------------------
    # 特征重要性分析方法
    # ---------------------
    # 置换特征重要性
    perm_importance = permutation_importance(model, X_test, y_test, scoring="roc_auc",
n_repeats=10, random_state=42)

    # 打印特征重要性
```

```
    print("置换特征重要性：", perm_importance.importances_mean)

    # 确保特征重要性有意义
    if np.any(perm_importance.importances_mean != 0):
        sorted_idx = perm_importance.importances_mean.argsort()
        plt.figure(figsize=(10, 8))
        plt.barh([top_features[i] for i in sorted_idx],
perm_importance.importances_mean[sorted_idx])
        plt.title("Permutation Feature Importance")
        plt.xlabel("Mean Decrease in AUC")
        plt.tight_layout()
        plt.savefig("permutation_importance.png", dpi=300, bbox_inches="tight")
        plt.show()
    else:
        print("置换特征重要性全为零，请检查模型输出或数据是否正常")
    X_test = scaler.inverse_transform(X_test)
    # 3. LIME 特征重要性
    lime_explainer = LimeTabularExplainer(
        X_train,
        training_labels=y_train,
        feature_names=top_features,
        mode="classification",
        random_state=42
    )

    # 选取测试集中的一个实例进行解释
    test_instance = X_test[0]
    lime_exp = lime_explainer.explain_instance(
        test_instance,
        model.predict_proba,
        num_features=10
    )

    # 绘制 LIME 特征重要性
    lime_exp.show_in_notebook(show_table=True)
    lime_exp.save_to_file("lime_importance.html")  # 保存为 HTML 文件
```
